# Sex differences in risk factors for depressive symptoms in patients with COPD: The 2014 and 2016 Korea National Health and Nutrition Examination Survey

**DOI:** 10.1186/s12890-021-01547-x

**Published:** 2021-05-28

**Authors:** Ji Soo Choi, Se Hyun Kwak, Nak-Hoon Son, Jae Won Oh, San Lee, Eun Hye Lee

**Affiliations:** 1 Division of Pulmonology, Allergy and Critical Care Medicine, Department of Internal Medicine, Yongin Severance Hospital, Yonsei University College of Medicine, 363 Dongbaekjukjeon-daero, Giheung-Gu, Yongin-si, Gyeonggi-do 16995 Republic of Korea; 2 Center for Digital Health, Yongin Severance Hospital, Yonsei University College of Medicine, Yongin, Gyeonggi-do Republic of Korea; 3 Data Science Team (Biostatistician), Center for Digital Health, Yongin Severance Hospital, Yonsei University College of Medicine, Yongin, Gyeonggi-do Republic of Korea; 4 Department of Psychiatry, Yongin Severance Hospital, Yonsei University College of Medicine, Yongin, Republic of Korea; 5 Department of Psychiatry and Institute of Behavioral Science in Medicine, Yonsei University College of Medicine, Seoul, Republic of Korea

**Keywords:** Depressive symptoms, Chronic obstructive pulmonary disease, Sex, Korea National Health and Nutrition Examination Survey (KNHANES)

## Abstract

**Background:**

Although depression is a common comorbidity of chronic obstructive pulmonary disease (COPD), the role of sex remains unexplored. We evaluated sex differences of risk factors of depressive symptoms in adults with COPD.

**Methods:**

This was a population-based cross-sectional study using data from the 2014 and 2016 Korea National Health and Nutrition Examination Survey. Spirometry was used to identify patients with COPD, defined as a FEV_1_/FVC ratio < 0.7. Presence of depressive symptoms was defined as a total score ≥ 5 on the Patient Health Questionnaire-9.

**Results:**

17.8% of participants expressed depressive symptoms. Relative regression analysis revealed that female sex (RR 2.38; 95% CI 1.55–3.66; p < 0.001), living alone (RR 1.46; 95% CI 1.08–1.97; p = 0.013), current smoker (RR 1.70; 95% CI 1.15–2.52; p = 0.008), underweight (RR 1.58 95% CI 1.00–2.49; p = 0.049), and GOLD Stage III/IV (RR 1.92; 95% CI 1.19–3.09; p = 0.007) were the risk factors for depressive symptoms. Low income, living alone, multiple chronic disorders, and low BMI were risk factors of depressive symptoms in male, whereas low educational attainment, urban living, and current smoking were risk factors in female.

**Conclusions:**

Female sex is a main risk factor of depressive symptoms in adults with COPD. As risk factors of depressive symptoms in COPD patients vary according to their sex, different approaches are needed to manage depression in males and females with COPD.

## Background

Chronic obstructive pulmonary disease (COPD) is characterized by airflow limitation resulting from airway inflammation and remodeling that is not fully reversible [1]. Patients with COPD frequently present with several comorbidities, including hypertension, cardiovascular disease, diabetes mellitus, osteoporosis, and lung cancer [2–5]. Depression is considered one of the most common comorbidities of COPD, with estimates of depression prevalence in COPD ranging from 10 to 57% [6]. Although depression in patients with COPD is related to a poor quality of life, more frequent exacerbations, and increase in mortality [5, 7, 8], previous studies reported that only 27–33% of those with depression were being treated for it [5, 9]. Untreated depressive symptoms may increase physical disability, morbidity, and healthcare utilization, while early identification of these psychiatric conditions and subsequent psychological intervention in patients with COPD might improve the clinical outcomes [7].

Previous studies have reported risk factors for depression in patients with COPD, including living alone, severe COPD, impaired physical functioning [10, 11]. However, studies on how the risk factors of depressive symptoms in COPD patients differ according to sex are insufficient. Therefore, the aim of this study was to identify that sex is a risk factor of depressive symptoms in patients with COPD. Furthermore, the secondary objective was to explore whether the risk factors of depressive symptoms were different in COPD patients according to sex.

## Methods

### Survey overview

The study data were obtained from the Korea National Health and Nutrition Examination Survey (KNHANES), a nationally representative population-based cross-sectional survey that evaluates the health and nutritional status of the Korean population conducted annually by the Centers for Disease Control and Prevention in Korea. The survey collected detailed information on demographic, socioeconomic, and clinical characteristics, including age, educational attainment, economic activity, household income, marital status, alcohol consumption, smoking habits, and previous and current diseases.

Survey participants were selected from 192 regions in Korea based on a stratified multistage sampling method. The survey was composed of several components, including a health behavior questionnaire, health interview, health examination, and nutritional survey. Participants aged over 40 years also performed a spirometry test using a spirometer as part of the health examination. The KNHANES 2014 and 2016 surveys included the Patient Health Questionnaire-9 (PHQ-9), which is a self-reported depression screening scale. Therefore, we used survey data from these two years. The KNHANES provides secondary data that is publicly available and a more detailed description of the survey profile can be found elsewhere [12].

### Study population

**Fig. 1:**
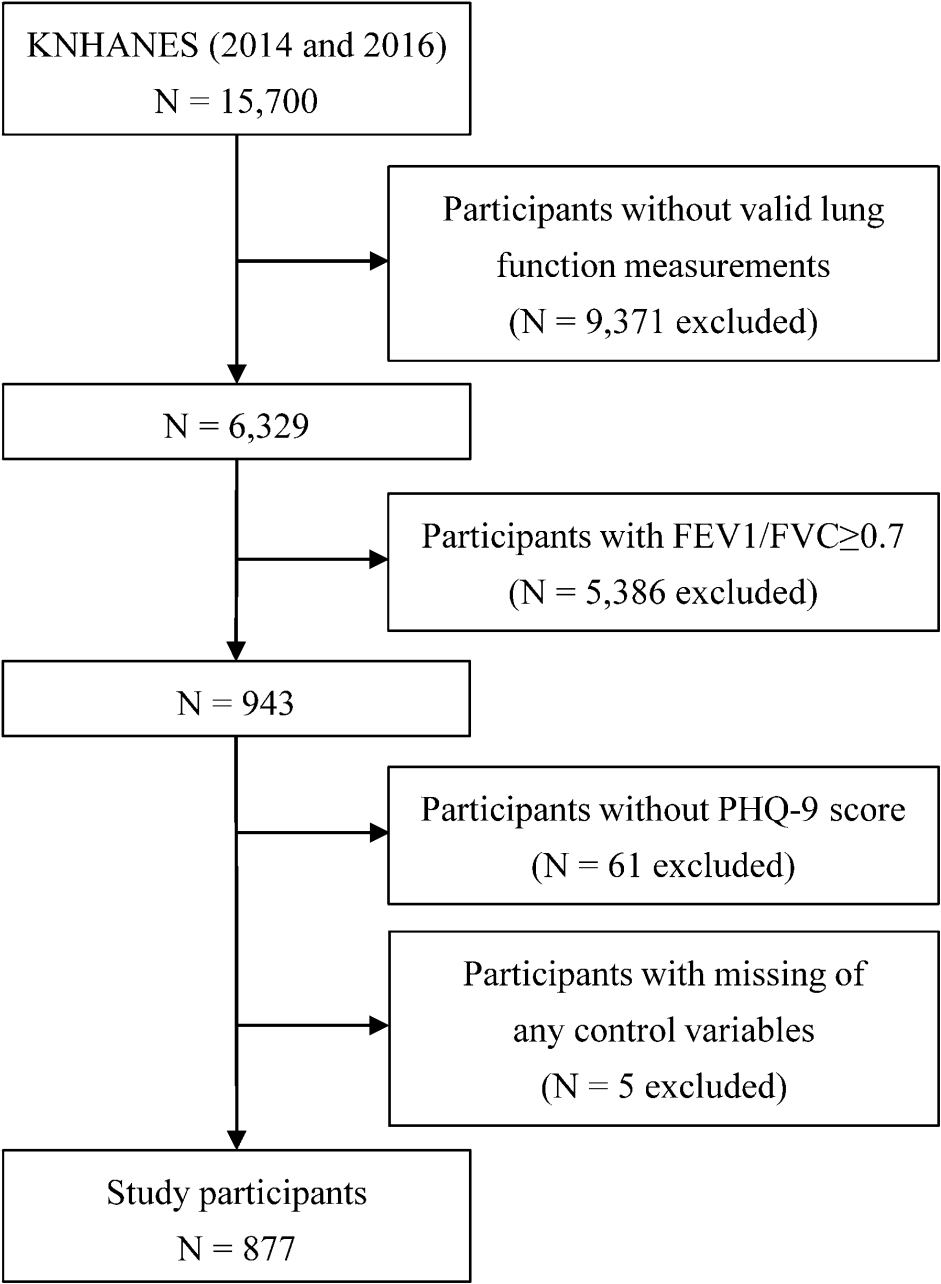
Flow diagram of the study participants. FEV1, forced expiratory volume in 1 s; FVC, forced vital capacity; KNHANES, Korea National Health and Nutritional Examination Survey; PHQ-9, Patient Health Questionnaire-9

This study initially assessed data from 6329 eligible participants with valid lung function measurements. After excluding participants who did not meet the COPD criteria, and those with any missing variables, including PHQ-9 score, a total of 877 participants were included in the final analysis (Fig. 1).

### Assessment of lung function

Lung function was measured by trained medical technicians according to the manual of the American Thoracic Society/European Respiratory Society Task Force, using dry rolling seal spirometers (Model 2130; Sensor Medics, Yorba Linda, CA, USA) [13]. The forced expiratory volume in 1 s (FEV1), forced vital capacity (FVC), and FEV1/FVC ratio were obtained. COPD was defined as a pre-bronchodilator FEV1/FVC ratio below 0.7, according to 2018 Global Initiative for Chronic Obstructive Lung Disease (GOLD) guidelines [14]. The severity of COPD (GOLD Stage) was classified based on the percent-predicted FEV1: Stage I (mild; FEV1 ≥ 80%), Stage II (moderate; FEV1 50–79%), Stage III (severe; FEV1 30–49%), and Stage IV (very severe; FEV1 < 30%).

### Assessment of body mass index and depressive symptoms

Body mass index (BMI) was calculated as weight/height squared (kg/m2) and classified into: underweight (BMI < 18.5), normal (18.5 ≤ BMI < 23), overweight (23 ≤ BMI < 25), and obese (BMI ≥ 25) according to the revised Asia–Pacific BMI criteria by the World Health Organization Western Pacific Region [15].

Depressive symptoms were assessed using the PHQ-9, a nine-item self-reported questionnaire [16]. The questionnaire is based on the criteria of the Diagnostic and Statistical Manual of Mental Disorders, Fourth Edition, which is used to diagnose several specific types of depressive disorders [17]. Each item on the PHQ-9 is scored on a scale from 0 to 3. The scores are then summed as a total score ranging between 0 and 27. PHQ-9 total scores of 5, 10, 15, and 20 represent valid thresholds of mild, moderate, moderately severe, and severe depression, respectively. PHQ-9 is in the public domain and the scale can be used free of charge [18]. Following previous studies on depressive symptoms using KNHANES data [19–21], the presence of depressive symptoms was defined as a PHQ-9 ≥ 5 in this study [22]. All participants were categorized into two groups depending on whether they had depressive symptoms according to this cut-off score.

### Assessment of covariates

We included socio-demographic variables: sex, age, educational attainment, equalized household income, living status, economic activity, and residential area. Age groups were divided into five categories as 40–49, 50–59, 60–69, 70–79, and 80 or older. Educational attainment was categorized as ‘elementary school or below,’ ‘middle school graduate,’ ‘high school graduate,’ or ‘college or above.’ Equalized household income was categorized into quartiles from ‘quartile 1 (low income)’ to ‘quartile 4 (high income).’ Living status was categorized as ‘living alone’ or ‘living together.’ Economic activity was categorized as ‘employed’ or ‘unemployed.’ Residential area was categorized as ‘urban’ or ‘rural.’

We also included health-related variables as additional potential confounders: alcohol consumption status, smoking status, chronic medical diseases, and GOLD Stage. Alcohol consumption and smoking status were categorized as ‘never use,’ ‘former use,’ or ‘current use.’ Chronic medical diseases, including hypertension, diabetes mellitus, angina, myocardial infarction, and stroke, were collected via self-reported doctor diagnosis. The total number of diseases was summed and classified into three groups as ‘zero,’ ‘one,’ and ‘two or more.’ For analyses, GOLD Stages III and IV were combined into one, resulting in three categories, ‘Stage I,’ ‘Stage II,’ and ‘Stage III/IV.’

### Statistical analysis

All data are presented as numbers and percentages. Categorical comparisons were performed using the chi-square test. Multivariable analysis was performed using the relative risk regression analysis with prespecified covariates. Relative risk (RR) and 95% confidence intervals (CIs) were also calculated. A p-value < 0.05 was considered significant for all analyses. Data analyses were performed using SAS software, version 9.4 (SAS Institute, Cary, NC, USA).

## Ethical approval

The research protocol was approved by the Institutional Review Board (IRB) of Severance Hospital. The need for informed consent was waived by the IRB of Severance Hospital as the data were de-identified in the database used (IRB number: 4–2019-0854).

## Results

### Baseline characteristics of the study participants

A total of 877 COPD participants were identified during the study period (Table 1), with a mean age at diagnosis of 66.1 years (SD 9.5) and a proportion of 71.5% males. Among these patients, 156 patients reported depressive symptoms, with an incidence of 17.8% (156/877).

**Table 1:** Characteristics of the study participants

	With depressive symptoms (PHQ-9 ≥ 5)	Without depressive symptoms (PHQ-9 < 5)	Total participants	P value
	(N = 156)	(N = 721)	(N = 877)	
*Sex*				**< 0.001**
Male	82 (52.6)	545 (75.6)	627 (71.5)	
Female	74 (47.4)	176 (24.4)	250 (28.5)	
Age (years, mean ± SD)	67.2 ± 9.5	65.9 ± 9.5	66.1 ± 9.5	0.125
*Age (years)*				0.336
40–49	9 (5.8)	54 (7.5)	63 (7.2)	
50–59	22 (14.1)	118 (16.4)	140 (16.0)	
60–69	50 (32.0)	259 (35.9)	309 (35.2)	
≥ 70	75 (48.1)	290 (40.2)	365 (41.6)	
*Educational attainment*				**< 0.001**
Elementary school or below	82 (52.6)	255 (35.4)	337 (38.4)	
Middle school	23 (14.7)	140 (19.4)	163 (18.6)	
High school	33 (21.2)	194 (26.9)	227 (25.9)	
College or above	18 (11.5)	132 (18.3)	150 (17.1)	
*Equalized household income*				**< 0.001**
Quartile 1 (low)	79 (50.6)	215 (29.8)	294 (33.5)	
Quartile 2	37 (23.7)	206 (28.6)	243 (27.7)	
Quartile 3	21 (13.5)	142 (19.7)	163 (18.6)	
Quartile 4 (high)	19 (12.2)	158 (21.9)	177 (20.2)	
*Living status*				**< 0.001**
Living together	102 (65.4)	614 (85.2)	716 (81.6)	
Living alone	54 (34.6)	107 (14.8)	161 (18.4)	
*Economic activity*				**0.006**
Employed	65 (41.7)	390 (54.1)	455 (51.9)	
Unemployed	91 (58.3)	331 (45.9)	422 (48.1)	
*Residential area*				0.176
Urban	69 (44.2)	274 (38.0)	343 (39.1)	
Rural	87 (55.8)	447 (62.0)	534 (60.9)	
*Alcohol consumption status*				**0.037**
Never drinker	26 (16.7)	95 (13.2)	121 (13.8)	
Former drinker	62 (39.7)	230 (31.9)	292 (33.3)	
Current drinker	68 (43.6)	396 (54.9)	464 (52.9)	
*Smoking status*				**< 0.001**
Never smoker	69 (44.3)	234 (32.5)	303 (34.6)	
Former smoker	40 (25.6)	310 (43.0)	350 (39.9)	
Current smoker	47 (30.1)	177 (24.5)	224 (25.5)	
*Chronic medical diseases*				0.465
None	76 (48.7)	364 (50.5)	440 (50.2)	
One	50 (32.1)	247 (34.2)	297 (33.9)	
Two or more	30 (19.2)	110 (15.3)	140 (15.9)	
BMI (kg/m^2^, mean ± SD)	23.3 ± 3.3	24.0 ± 2.9	23.8 ± 3.0	**0.024**
*BMI*				**< 0.001**
Underweight	12 (7.7)	14 (1.9)	26 (3.0)	
Normal weight	62 (39.7)	258 (35.8)	320 (36.5)	
Overweight	34 (21.8)	198 (27.5)	232 (26.4)	
Obesity	48 (30.8)	251 (34.8)	299 (34.1)	
*GOLD stage*				**0.019**
Stage 1	75 (48.1)	373 (51.7)	448 (51.1)	
Stage 2	67 (43.0)	321 (44.5)	388 (44.2)	
Stage 3 and 4	14 (8.9)	27 (3.8)	41 (4.7)	

Data are presented as numbers (%) or mean ± SD

A p value of < 0.05 was considered significant (bold) for all analyses

PHQ-9, patient health questionnaire-9; BMI, body mass index; SD, standard deviation; GOLD, global initiative for chronic obstructive lung disease

There was a higher proportion of females among those who’ve reported depressive symptoms in comparison to those without depressive symptoms. Low educational attainment, low household income, current smoking, underweight, and GOLD Stage III/IV were also found to be associated with depressive symptoms. Detailed description is available in Table 1.

### Factors associated with depressive symptoms

Table 2 shows the results of relative risk regression analysis for the associated factors with depressive symptoms. According to the adjusted multiple regression analysis, the following variables were associated with depressive symptoms; female sex (RR 2.38; 95% CI 1.55–3.66; p < 0.001), living alone (RR 1.46; 95% CI 1.08–1.97; p = 0.013), current smoker (RR 1.70; 95% CI 1.15–2.52; p = 0.008), underweight (RR 1.58 95% CI 1.00–2.49; p = 0.049), and GOLD Stage III/IV (RR 1.92; 95% CI 1.19–3.09; p = 0.007). Furthermore, there was a 43% reduction in the incidence of depressive symptoms (RR 0.57; 97.5% CI 0.33–0.97; P = 0.039) among high household income participants.

**Table 2:** Results of the relative risk regression analysis for the associated factors with depressive symptoms (PHQ-9 ≥ 5)

	RR	95% CI		P value
*Sex*				
Male	1.00			
Female	**2.38**	**1.55**	**3.66**	** < 0.001**
*Age (years)*				
40–49	1.00			
50–59	0.95	0.45	1.98	0.886
60–69	0.88	0.43	1.80	0.728
≥ 70	0.89	0.42	1.88	0.750
*Educational attainment*				
Elementary school or below	1.00			
Middle school	0.73	0.47	1.14	0.167
High school	0.76	0.50	1.15	0.200
College or above	0.80	0.46	1.39	0.426
*Equalized household income*				
Quartile 1 (low)	1.00			
Quartile 2	0.79	0.54	1.16	0.234
Quartile 3	0.67	0.42	1.08	0.104
Quartile 4 (high)	**0.57**	**0.33**	**0.97**	**0.039**
*Living status*				
Living together	1.00			
Living alone	**1.46**	**1.08**	**1.97**	**0.013**
*Economic activity*				
Employed	1.00			
Unemployed	1.06	0.78	1.45	0.695
*Residential area*				
Urban	1.00			
Rural	0.78	0.59	1.03	0.080
*Alcohol consumption status*				
Never drinker	1.00			
Former drinker	1.08	0.72	1.63	0.713
Current drinker	1.09	0.71	1.69	0.688
*Smoking status*				
Never smoker	1.00			
Former smoker	1.23	0.75	2.02	0.412
Current smoker	**1.70**	**1.15**	**2.52**	**0.008**
*Chronic medical diseases*				
None	1.00			
One	0.91	0.65	1.28	0.598
Two or more	1.01	0.68	1.51	0.947
*BMI*				
Underweight	**1.58**	**1.00**	**2.49**	**0.049**
Normal weight	1.00			
Overweight	0.75	0.51	1.10	0.145
Obesity	0.82	0.58	1.17	0.275
*GOLD stage*				
Stage 1	1.00			
Stage 2	0.99	0.74	1.34	0.957
Stage 3 and 4	**1.92**	**1.19**	**3.09**	**0.007**

A p value of < 0.05 was considered significant (bold) for all analyses

PHQ-9, Patient health questionnaire-9; BMI, body mass index; GOLD, global initiative for chronic obstructive lung disease; RR, relative risk; CI, confidence interval

### Sex differences in the factors associated with depressive symptoms

Multivariable relative regression analysis was used to compare sex stratified models that estimated the RRs of depressive symptoms (Table 3). Male with lower household income, living alone (RR 1.97; 95% CI 1.18–3.28; p = 0.009), with the presence of two or more chronic medical diseases (RR 2.04; 95% CI 1.15–3.63; p = 0.015), and low BMI (RR 3.35; 95% CI 1.16–9.68; p = 0.026) showed association with depressive symptoms. For females, low educational attainment, living in urban, and current smoking were significant factors associated with depressive symptoms. Detailed description is available in Table 3.

**Table 3:** Results of the relative risk regression analysis for the associated factors with depressive symptoms (PHQ-9 ≥ 5) stratified by sex

	Male				Female			
	N = 627				N = 250			
	RR	95% CI		P value	RR	95% CI		P value
*Age (years)*								
40–49	1.00				1.00			
50–59	1.10	0.39	3.07	0.861	0.75	0.23	2.42	0.627
60–69	1.26	0.49	3.23	0.634	0.48	0.14	1.66	0.244
≥ 70	0.98	0.36	2.61	0.960	0.76	0.20	2.81	0.677
*Educational attainment*								
Elementary school or below	1.00				1.00			
Middle school	0.94	0.50	1.77	0.851	0.50	0.23	1.09	0.080
High school	1.49	0.87	2.55	0.151	**0.28**	**0.11**	**0.69**	**0.006**
College or above	1.84	0.93	3.63	0.078	**0.24**	**0.07**	**0.84**	**0.025**
*Equalized household income*								
Quartile 1 (low)	1.00				1.00			
Quartile 2	**0.50**	**0.29**	**0.89**	**0.017**	1.39	0.79	2.44	0.254
Quartile 3	**0.53**	**0.28**	**0.98**	**0.041**	0.76	0.33	1.74	0.510
Quartile 4 (high)	**0.24**	**0.10**	**0.57**	**0.001**	1.37	0.69	2.71	0.368
*Living status*								
Living together	1.00				1.00			
Living alone	**1.97**	**1.18**	**3.28**	**0.009**	1.32	0.81	2.16	0.269
*Economic activity*								
Employed	1.00				1.00			
Unemployed	0.86	0.54	1.38	0.534	1.36	0.83	2.24	0.223
*Residential area*								
Urban	1.00				1.00			
Rural	0.97	0.64	1.46	0.889	**0.56**	**0.36**	**0.88**	**0.012**
*Alcohol consumption status*								
Never drinker	1.00				1.00			
Former drinker	1.71	0.71	4.15	0.234	1.12	0.65	1.93	0.677
Current drinker	1.59	0.67	3.80	0.295	1.06	0.51	2.20	0.874
*Smoking status*								
Never smoker	1.00				1.00			
Former smoker	0.91	0.44	1.88	0.805	1.78	0.54	5.91	0.342
Current smoker	1.56	0.76	3.21	0.228	1.51	0.76	2.99	0.233
*Chronic medical diseases*								
None	1.00				1.00			
One	1.36	0.84	2.20	0.212	0.64	0.35	1.16	0.138
Two or more	**2.04**	**1.15**	**3.63**	**0.015**	0.56	0.27	1.15	0.113
*BMI*								
Underweight	**3.35**	**1.16**	**9.68**	**0.026**	1.41	0.58	3.45	0.445
Normal weight	1.00				1.00			
Overweight	0.99	0.58	1.67	0.968	0.66	0.33	1.29	0.219
Obesity	0.89	0.54	1.46	0.640	0.61	0.32	1.13	0.113
*GOLD stage*								
Stage 1	1.00				1.00			
Stage 2	0.70	0.45	1.09	0.115	1.37	0.82	2.26	0.225
Stage 3 and 4	1.53	0.78	2.99	0.214	2.50	0.89	7.08	0.083

A p value of < 0.05 was considered significant (bold) for all analyses

PHQ-9, patient health questionnaire-9; BMI, body mass index; GOLD, global initiative for chronic obstructive lung disease; RR, relative risk; CI, confidence interval

## Discussion

In this study, we investigated the prevalence and risk factors of depressive symptoms in adults with COPD, finding that the incidence of depressive symptoms was 17.8% (156/877). Female sexwas a main risk factor of depressive symptoms in adults with COPD. Additionally, lower BMI, living alone, being a current smoker, and having a high severity of COPD (GOLD Stage III/IV) were significant risk factors of depressive symptoms. Furthermore, we found that the risk factors of depressive symptoms in COPD patients were different with respect to sex. In males, low BMI, low income, living alone, and combined chronic medical disease were related with the occurrence of depressive symptoms, whereas low educational attainment, living in an urban setting, and being a current smoker were risk factors in females.

Depression is common in patients with chronic disease, including COPD, heart disease, stroke, and diabetes, and screening and providing interventions for depression early should be emphasized to prevent worsening of the disease [23]. In COPD patients specifically, depression is one of the most common comorbidities with a prevalence of 10–57% [6], and is related to a higher risk of acute exacerbation, frequent hospitalization, and mortality [24, 25]. Therefore, early detection of depression followed by proper interventions in COPD patients are important to control depressive symptoms and clinical prognosis in COPD patients [26].

The PHQ-9 is a brief version, composed of nine items, of a longer depression scale, the Patient Health Questionnaire (PHQ), that is based on the DSM-IV diagnosis of depressive disorders [27]. This brief screening instrument can easily be used in primary healthcare centers and is considered to have comparable sensitivity and specificity to other depression screening instruments [7]. Many studies have been conducted to determine an appropriate cut-off value of the PHQ-9 when screening for depression [28], with some reporting that a cut-off value over 10, which is classified as moderate depression severity on the scale, is considered to be reliable for screening for depressive disorders [29, 30]. However, Lesley et al. reported that PHQ-9 ≥ 5 was the optimal cut-off value to detect depression in patients with coronary artery disease, which was lower than even the recommended cut-off score [31]. Likewise, Han et al. also recommended that a PHQ-9 score of 5, based on the Korean version, was appropriate to detect depression in elderly patients [22]. In our study, 76.9% (674/877) of participants were elderly patients over 60 years old. Moreover, the prevalence of depression in our participants with COPD was 17.8%, which is similar to other previous studies that also defined depressive symptoms based on a PHQ-9 score of ≥ 5. Therefore, in this study, we chose this value to define the presence of depressive symptoms. While this value may be lower than those in previous reports, the benefit is that we may be able to detect depressive symptoms in COPD patients earlier and initiate the proper intervention.

In previous studies, the severity of COPD, living alone, respiratory symptoms [10], and lower BMI [32] were significantly related to the development of depression in patients with COPD. Additionally, Jasmin et al. found that factors including female sex, low socioeconomic status, lower FEV1, the degree of dyspnea, smoking, obesity, low social support, and loneliness were associated with an increase of depression in obstructive lung disease [11]. In the current study, female sex, low BMI, living alone, being a current smoker, and having a GOLD Stage III/IV were also significant risk factors for depressive symptoms. However, low income, educational attainment, and household income were not significantly different between patients with COPD comorbid with depression.

COPD is no longer a disease more commonly found in males owing to increased tobacco use in females and exposure to air pollution [33]. It has been demonstrated that the susceptibility to COPD, related risk factors, clinical presentation, comorbidities, and response to treatment differ between males and females [34]. Among them, depression and other psychological disorders, including anxiety and irritability, are more prevalent in females [35]. Therefore, greater effort should be made in identifying and managing these conditions [36]. In a subgroup analysis, we found that there were differences in the risk factors of depression in COPD patients by sex, indicating that different approaches may be needed to predict depression in COPD patients depending on their sex.

This study has several limitations. The first is that this was a retrospective study based on data from the KNHANES, a nationwide survey that evaluated the health and nutritional status of the Korean population. Hence, it is possible that COPD patients with moderate to high severity were not included, as this data included the results of healthy patients for medical screening. However, our findings could help in detecting depressive symptoms early and prevent disease progression in mild COPD patients. Second, we analysed using only pre-bronchodilator results because post-bronchodilator results were not available in the KNHANES protocol. However, the COPD patients with only pre-bronchodilator results and no post-bronchodilator results had shown fairly similar presentation when compared to patients with both pre-bronchodilator and post-bronchodilator results [37]. Third, the severity of symptoms and the treatment the patients received, aspects that could affect the patients’ quality of life and the development of depression, were not able to be investigated. Further study regarding this point should be undertaken to support our findings.

Nevertheless, our research also has its strengths, in that it shows the risk factors associated with the occurrence of depressive symptoms in COPD patients, including a difference in risk factors between males and females. Furthermore, we demonstrated that there is an opportunity to detect and manage depressive symptoms at an early stage by analyzing the data of patients with mild severity of COPD, though prospective and large-sample studies are needed for validation.

## Conclusions

We found that the incidence of depressive symptoms was not lower even in patients with mild COPD. However, female sex was a main risk factor for depressive symptoms in adults with COPD. Thus, the risk factors of depressive symptoms in COPD patients differed according to sex. We suggest that COPD patients found to have these risk factors should be kept under close observation to prevent depression and exacerbation of disease-related symptoms.

## Data Availability

This study analyzed data from the 2014 and 2016 KNHAES. All the KNHANES data are available to the public and can be downloaded from the KNHANES official website (http://knhanes.cdc.go.kr/).

## Abbreviations

COPD: Chronic obstructive pulmonary disease
KNHANES: Korea National Health and Nutrition Examination Survey
PHQ-9: Patient Health Questionnaire-9
FEV1: Forced expiratory volume in 1 s
FVC: Forced vital capacity
GOLD: Global Initiative for Chronic Obstructive Lung Disease
BMI: Body mass index
CIs: Confidence intervals
ORs: Odds ratios
